# Complete Genome Sequence of Escherichia Phage 590B, Active against an Extensively Drug-Resistant Uropathogenic Escherichia coli Isolate

**DOI:** 10.1128/MRA.00550-21

**Published:** 2021-09-23

**Authors:** Naveen Chaudhary, Dharminder Singh, Chandradeo Narayan, Bhaskar Samui, Balvinder Mohan, Ravimohan S. Mavuduru, Neelam Taneja

**Affiliations:** a Department of Medical Microbiology, Postgraduate Institute of Medical Education and Research, Chandigarh, India; b Department of Urology, Postgraduate Institute of Medical Education and Research, Chandigarh, India; Loyola University Chicago

## Abstract

Escherichia phage 590B, which was isolated from community sewage water in Chandigarh, India, exhibited lytic activity against an extensively drug-resistant uropathogenic Escherichia coli isolate. The genome of the phage is linear, double stranded, and 44.39 kb long. Phage 590B is a member of the *Siphoviridae* family and is closest to phage vB_EcoS_XY2, which was isolated in China.

## ANNOUNCEMENT

Urinary tract infections (UTIs) are very common in both community and hospital settings (1). A majority of UTIs are caused by uropathogenic Escherichia coli (UPEC) (2). Treatment of UTIs has become challenging due to the emergence of highly drug-resistant strains (3). Intravesical bacteriophage therapy is an attractive alternative to antibiotics in such clinical settings.

Here, we report the complete genome sequence of Escherichia phage 590B, which we found to be active against an extensively drug-resistant UPEC strain. The phage was isolated from raw sewage water of a community treatment plant in Chandigarh, India, using broth culture of host bacterial isolate UPEC 590, which is resistant to third-generation cephalosporins, amikacin, gentamicin, nitrofurantoin, nalidixic acid, ciprofloxacin, imipenem, meropenem, co-trimoxazole, tazobactam-piperacillin, and cefoperazone-sulbactam. For phage isolation, 0.5 ml bacterial broth culture (10^8^ CFU/ml) was mixed with 3 ml raw sewage water and incubated at 37°C for 24 h. A single isolated phage plaque (<4 mm in diameter) with clear morphology was picked and transferred to SM buffer (5.8 g/liter NaCl, 100 mM MgSO_4_·7H_2_O). This step was repeated three times to obtain isolated phage plaques in a soft agar plaque assay. The phage was amplified by incubating the host bacterial culture (UPEC 590) with phage at a multiplicity of infection (MOI) of 1 for 24 h at 37°C. Ultracentrifugation was performed at 50,000 rpm for 2 h, and the pellet was resuspended in SM buffer (4). DNA was extracted using a phage DNA isolation kit (Norgen Biotek, Canada). DNA quality was measured using a Qubit 3.0 fluorometer (Promega, USA). The phage sequencing library was prepared with the NEBNext Ultra library preparation kit, and sequencing was executed on an Illumina HiSeqX Ten sequencer, producing 1,893,036 paired-end (150-bp-long) raw reads with an average depth of 4,000×. The FASTQ files were preprocessed with Trimmomatic and TrimGalore (5, 6). The adapter sequences were filtered out with AdapterRemoval v2 (7). Iterative Virus Assembler (IVA) v1.0.8 was used to perform *de novo* assembly, and the default k-mer sizes were used (8). Phage open reading frames (ORFs) were predicted using GeneMarkS v4.28 and GLIMMER v3.02 (9). The assembled genome of bacteriophage 590B was annotated with Rapid Annotation using Subsystem Technology (RAST) v2.0 and verified individually by NCBI BLAST. We screened the complete phage genome against the Virulence Factor Database (VFDB) 2019, the Comprehensive Antibiotic Resistance Database (CARD) with Resistance Gene Identifier (RGI) v5, and ARAGORN v1.2.36 to find virulence factors, antibiotic resistance genes, and tRNAs, respectively (10–12). All software and tools were used with default parameters and settings. The work was approved by the Institute Ethical Clearance Committee of the Postgraduate Institute of Medical Education and Research (Chandigarh, India).

IVA generated a single contig of 44.39 kb with a GC content of 50.89% and a gene density of 1.17/kbp. Fifty-two ORFs were predicted, and the putative functions of the related ORFs were confirmed using BLASTp against the NCBI nonredundant database. Twenty-nine ORFs (55.7%) and 23 ORFs (44.2%) were predicted to encode functional proteins and hypothetical proteins, respectively. ORF 41 (nucleotide positions 39903 to 40391) encoded phage endolysin (EC 3.2.1.17), which is a highly specific hydrolytic enzyme of tailed phages that cleaves peptidoglycan, the major component of the host bacterial cell wall (13).

A BLASTn similarity analysis showed that the 590B genome is closest (query coverage, 92%; identity, 94.35%) to Escherichia phage vB_EcoS_XY2 (GenBank accession no. MN927226.1); this phage belongs to the *Siphoviridae* family of noncontractile long-tailed phages, which comprises approximately 61% of bacterial viruses (14, 15). In conventional phage therapy, strictly lytic phages that obligately lyse or kill their bacterial host isolates are required. Escherichia phage 590B formed clear plaques and does not carry genes associated with tRNAs, antibiotic resistance, integrase, recombinase, or virulence factors. In conclusion, we have isolated a lytic phage that is active against an extremely drug-resistant UPEC isolate and can be a potential therapeutic candidate for UTIs.

### Data availability.

The complete genome sequence of Escherichia phage 590B was deposited in NCBI GenBank (accession no. MW722821). The raw sequencing reads are available at SRA accession no. SRR13926753, BioSample accession no. SAMN17915100, and BioProject accession no. PRJNA701846.
